# Accelerated and increased joint damage in young mice with global inactivation of mitogen-inducible gene 6 after ligament and meniscus injury

**DOI:** 10.1186/ar4522

**Published:** 2014-03-27

**Authors:** Danese M Joiner, Kennen D Less, Emily M Van Wieren, Yu-Wen Zhang, Daniel Hess, Bart O Williams

**Affiliations:** 1 Center for Skeletal Disease and Tumor Metastasis, Van Andel Research Institute, 333 Bostwick Avenue NE, Grand Rapids, MI 49503, USA; 2 Center for Cancer and Cell Biology, Van Andel Research Institute, 333 Bostwick Avenue NE, Grand Rapids, MI 49503, USA; 3 Michigan State University College of Human Medicine, 15 Michigan Street NE, Grand Rapids, MI 49503, USA; 4 Division of Pulmonary & Critical Care Medicine, University of Michigan, Biomedical Science Research Building, 109 Zina Pitcher Place Room 4062, Ann Arbor, MI 48109-2200, USA

## Abstract

**Introduction:**

Ligament and meniscal damage can cause joint disease. Arthritic joints contain increased amounts of epidermal growth factor receptor (EGFR) protein, and polymorphisms in *EGFR* are associated with arthritis risk. The role of endogenous EGFR regulation during joint disease due to ligament and meniscal trauma is unknown. Mitogen-inducible gene 6 (*MIG-6*) can reduce EGFR phosphorylation and downstream signaling. We examined the effect of EGFR modulation by *MIG-6* on joint disease development after ligament and meniscus injury.

**Methods:**

Knee ligament transection and meniscus removal were performed surgically on mice homozygous for a global inactivating mutation in MIG-6 (*Mig-6*^*−/−*^) and in wild-type (WT) animals.

**Results:**

Two weeks after surgery, *Mig-6*^*−/−*^mice had bone erosion as well as greater fibrous tissue area and serum RANKL concentration than WT mice. Four weeks after surgery, *Mig-6*^*−/−*^mice had less cartilage and increased cell proliferation relative to contralateral control and WT knees. Increased apoptotic cells and growth outside the articulating region occurred in *Mig-6*^*−/−*^mice. Tibia trabecular bone mineral density (BMD) and the number of trabeculae were lower in surgically treated knees relative to the respective control knees for both groups. BMD, as well as trabecular thickness and number, were lower in surgically treated knees from *Mig-6*^*−/−*^mice relative to WT surgically treated knees. Phosphorylated EGFR staining in surgically treated knees decreased for WT mice and increased for *Mig-6*^*−/−*^mice. Fewer inflammatory cells were present in the knees of WT mice.

**Conclusion:**

*Mig-6*^*−/−*^mice have rapid and increased joint damage after ligament and meniscal trauma. *Mig-6* modification could lessen degenerative disease development after this type of injury.

## Introduction

Degenerative joint disease is painful and debilitating for millions of individuals. Such disease is typically classified as osteoarthritis (OA) or rheumatoid arthritis (RA). OA is characterized by progressive loss of articular cartilage, formation of bony outgrowths (osteophytes) and loss of mobility [1]. During OA, the extracellular matrix (ECM) network of cartilage tissue degrades, water content increases, the size of ECM molecules decreases, the structure of the collagen network is damaged and chondrocytes attempt to compensate through increased proliferation and metabolism [2]. OA chondrocytes proliferate and cluster as well as increase the production of both matrix proteins and matrix-degrading enzymes [3]. Decreased chondrocyte metabolism and an inability to adequately repair and regenerate the damaged tissue lead to complete loss of cartilage tissue, subchondral bone fibrillation and cyst formation [2,4,5].

The results of work by other researchers also suggest that ECM mutations and disruption, as well as alterations in the mechanotransductory sensory feedback loop, which translates extracellular information to the cell to facilitate the secretion of ECM, may contribute to OA pathogenesis [6,7]. Pathologic cellular and structural changes in ligaments, the supporting musculature and fibrocartilaginous structures such as the meniscus and the synovium (that is, synovial lining hyperplasia, macrophage and lymphocyte infiltration, neoangiogenesis, and fibrosis) are also observed in OA [8]. RA is characterized by the presence of an immune-mediated inflammatory synovitis that can invade and destroy the ECMs of joint cartilage and bone [9]. Currently, there is no preventative or regenerative therapy for degenerative joint disease and in an effort to advance treatment, research has been focused on identifying the cell signaling pathways involved in arthritic disease development [3].

The epidermal growth factor receptor (EGFR) signaling pathway plays important roles in postnatal somatic growth, chondrocyte differentiation and bone metabolism [10-14]. EGFR is a tyrosine kinase receptor that binds to a family of epidermal growth factor (EGF)–like ligands, including EGF, amphiregulin, transforming growth factor α (TGFα), heparin-binding EGF (HB-EGF), betacellulin (BTC) and epiregulin. Upon ligand binding, EGFR activates intracellular signal transduction pathways (including Ras/Raf/mitogen-activated protein kinase (MAPK) and phosphoinositide 3-kinase (PI3K)/Akt) in a variety of cells, thus modulating proliferation, survival, adhesion, migration and differentiation [11].

Mice with osteoblast-specific deletion of EGFR have a low bone mass phenotype, whereas mice with high EGFR activity have a high bone mass phenotype [13]. Inhibited EGFR signaling can alter endochondral ossification in mice and rats, and EGFR global knockout (KO) mice display increased chondrocyte hypertrophy [14,15]. TGFα signaling controls endochondral ossification during bone growth [16]. EGFR signaling is involved in diseases that affect cartilage. In humans, there is a significant increase in the risk of RA development with the occurrence of the single-nucleotide polymorphism rs17337023 in *EGFR*, and arthritic joints have increased EGFR2 protein in the synovial fluid and membrane [17,18]. Pharmacological inhibition of EGFR can reduce the severity of collagen-induced RA in mice, and the inhibition of EGFR by adenovirus can reduce joint damage in arthritic mice [19,20]. Increased TGFα and EGFR signaling have also been shown in rat OA [21,22].

Little is known about the influence of endogenous EGFR regulation on skeletal diseases. Exogenous biglycan can regulate the expression of EGFR in human chondrocytes, and mitogen-inducible gene 6 (*MIG-6*)—also known as Gene 33, ErbB receptor feedback inhibitor 1 (*ERRFI1*) and *RALT*—is a negative feedback regulator of EGFR [23,24]. The MIG-6 protein is a nonkinase scaffolding adaptor found in the cytosol. When transcriptionally induced by EGF, *MIG-6* can reduce EGFR tyrosine phosphorylation and thus also reduce downstream extracellular signal-regulated kinase (ERK), c-Jun N-terminal kinase (JNK) and protein kinase B (Akt) activation through direct interaction with the EGFR family [24,25]. *MIG-6* is an immediate early response gene whose expression can be rapidly and robustly induced under normal and pathological conditions by hormones, growth factors and stress [24]. Reports of downregulated *MIG-6* expression in a rat surgical meniscectomy OA knee model and spontaneous development of an OA-like phenotype in 3-month-old mice with a global Mig-6 KO inactivating mutation (*Mig-6*^*−/−*^) suggest a role for *MIG-6* in joint disease. The influence of endogenous EGFR regulation on joint disease progression after trauma to ligaments and menisci has been unexplored [26,27].

Ligament and meniscus injury can alter mechanical loads placed on the joint and cause severe damage to the osteochondral joint surface and underlying trabecular bone, thus contributing to the onset and progression of joint disease [28-30]. We used a ligament/meniscus transection model in *Mig-6*^*−/−*^mice to investigate the endogenous regulation of EGFR by *MIG-6* and its effects on cartilage and bone damage after severe trauma. The identification of early signaling cascades and knowledge of cell behavior after ligament and meniscus injury are vital to developing therapies for subsequent arthritis development and progression. Therefore, we examined the early effects of ligament and meniscus injury on bone and cartilage in *Mig-6*^*−/−*^and wild-type (WT) mice. We hypothesized that global KO of *Mig-6* in mice would increase EGFR signaling and disrupt cartilage and subchondral bone homeostasis, leading to severe damage to the cartilage and subchondral bone after ligament and meniscus injury. Furthermore, we sought to determine whether untreated ligament and meniscus injury would rapidly progress to severe degenerative joint disease in *Mig-6*^*−/−*^mice.

## Materials and methods

### Ethical approval

The Van Andel Research Institute Institutional Animal Care and Use Committee approved all experimental procedures.

Generation of mice and surgical destabilization model

*Mig-6*^*−/−*^mice were generated as described previously [26]. The percentage of *Mig-6*^*−/−*^mice alive at birth was less than that of heterozygous and WT littermates (6% of *Mig-6*^*−/−*^, 65% of *Mig-6*^*+/−*^and 29% of WT mice); therefore, some *Mig-6*^*−/−*^sample groups were smaller than the corresponding WT groups for experimental assays [31]. Transection of the cruciate and meniscotibial ligaments, as well as meniscus removal, were performed on the right knees of 4-week-old *Mig-6*^*−/−*^mice (*n* = 36) and their 4-week-old WT littermates (*n* = 42) using an adaptation of a previously described surgical method [32].

Mice were anesthetized by intraperitoneal injection of 0.3 ml/g tribromoethanol and received 0.1 ml of ketoprofen. The right medial meniscotibial ligament was sectioned to destabilize the medial meniscus. The anterior cruciate, posterior cruciate and lateral meniscotibial ligaments were transected, and the medial and lateral menisci were removed. The patellar tendon and collateral ligaments were not transected. The incision was then closed with sutures and stapled. The animals resumed normal ambulation within 1 - 2 hours after surgery. *Mig-6*^*−/−*^mice showed spontaneous joint space narrowing at 1.5 months of age and increased joint damage severity at 3 months of age. Therefore, surgery was performed prior to spontaneous disease onset on 4-week-old mice. Mice were killed either 2 or 4 weeks after surgery by CO_2_ inhalation for endpoint analyses to examine the influence of injury prior to observation of significant spontaneous disease. On the basis of the limited number of *Mig-6*^*−/−*^mice per litter and the survival rate of *Mig-6*^*−/−*^animals after surgery, we used the left knee from each animal as an untouched contralateral control to acquire the sample sizes we needed for the outcome measures. Other investigators have found negligible cartilage damage similar to WT age-matched controls in the contralateral joints of mice subjected to surgical destabilization [33].

### *In vivo* X-ray and micro–computed tomography

WT (*n* = 3) and *Mig-6*^*−/−*^ (*n* = 3) mice were anesthetized with 2.5% isoflurane in oxygen and X-rayed every week after surgery with a piXarray digital specimen radiography system (Bioptics Faxitron, Tucson, AZ, USA). Knees from WT mice (*n* = 10) and *Mig-6*^*−/−*^mice (*n* = 6) were imaged after excision at 28 days after surgery using a SKYSCAN 1172 micro-CT scanner (Bruker, Ettlingen, Germany). Samples were immersed in phosphate-buffered saline and scanned, and damage to the knee joint trabecular bone was analyzed according to established laboratory protocols [34].

### Histology

Knees from WT mice (*n* = 17) and *Mig-6*^*−/−*^mice (*n* = 14) were fixed and decalcified. Paraffin-embedded sections (5 μm) were taken from the center of the medial condyle through the center of the lateral condyle. Sagittal sections were stained with hematoxylin and eosin and Movat’s pentachrome according to standard laboratory protocols in order to examine cartilage and subchondral bone. Nine sections from each sample were used for each stain. The greatest perpendicular distance of articular cartilage thickness was measured with ImageJ software (US National Institutes of Health, Bethesda, MD, USA). Three physicians blinded to the samples scored histological sections according to the Osteoarthritis Research Society International scoring scale [35]. The lowest score of 0 on this scale corresponds to normal tissue and the highest score of 6 corresponds to bone remodeling and microfibrocartilaginous/osseous repair. The three grades for each section were averaged, and the data from each group of mice were combined.

EGFR signaling is important for osteoclastogenesis; therefore, tartrate-resistant acid phosphatase (TRAP) staining was performed with a kit obtained from Sigma-Aldrich (St Louis, MO, USA) according to manufacturer’s instructions to detect osteoclasts [36]. A terminal deoxynucleotidyl transferase deoxyuridine triphosphate nick-end labeling (TUNEL) assay was performed according to the manufacturer’s instructions to assess cellular apoptosis (Promega, Madison, WI, USA). Immunohistochemistry (IHC) analysis was performed with a VECTASTAIN ABC Kit (Vector Laboratories, Burlingame, CA, USA) for Ki67 (SB M3060 at 1:100 dilution), a marker for cell proliferation. We also performed IHC on sections for monocytes, neutrophils and macrophages (prediluted ab75693; the immunogen for this product is an affinity-purified monocyte membrane preparation) according to the manufacturer’s instructions. IHC negative controls were incubated in blocking buffer instead of primary antibody. Stains were imaged with a Nikon Eclipse 55i microscope equipped with a Nikon Digital Sight camera (Nikon Instruments, Melville, NY, USA). The percentage of pixels that stained positive for Ki67, as well as monocytes, neutrophils and macrophages, was quantified using a Nuance system (Burlington, MA, USA). Quantitative stain analysis was performed on sections taken from the same-depth regions of the knee joints in WT and KO mouse control and surgically treated knees. Immunofluorescence (IF) was performed on sections for phospho-EGFR (1:800) and total EGFR (1:50) according to the manufacturer’s instructions. IF images were obtained using an EVOS system (Advanced Microscopy Group, Bothell, WA, USA), and all antibodies were purchased from Abcam (Cambridge, MA, USA), Cell Signaling Technology (Beverly, MA, USA), Spring Bioscience (Pleasanton, CA, USA) or EMD Millipore (Billerica, MA, USA).

### Quantitative RT-PCR

Knees from WT mice (*n* = 8) and *Mig-6*^*−/−*^mice (*n* = 7) were dissected under RNase-free conditions and homogenized in 1 ml of TRIzol reagent (Life Technologies, Carlsbad, CA, USA) with a Fast Prep-24 tissue and cell homogenizer (MP Biomedicals, Solon, OH, USA). RNA was chloroform-precipitated and extracted with an RNeasy Mini Kit (QIAGEN, Valencia, CA, USA) according to the manufacturer’s instructions. RNA concentration was calculated and integrity was analyzed using a NanoDrop 2000 spectrophotometer (Thermo Scientific, Wilmington, DE, USA) and an Agilent 2100 Bioanalyzer (Agilent Technologies, Santa Clara, CA, USA), respectively, and potential DNA contamination was removed by RNase-free DNase treatment (amplification grade DNaseI; Sigma-Aldrich). cDNA was made with a RevertAid First Strand cDNA Synthesis Kit (Fermentas, Glen Burnie, MD, USA) on 0.5 μg of RNA. Primers used for markers of osteogenesis and chondrogenesis (Integrated DNA Technologies, Coralville, IA, USA) were as follows: *sox9*: 5′- GCAGACCAGTACCCGCATCT-3′ and 5′-CTCGTTCAGCAGCCTCCAG-3′; *aggrecan*: 5′*-* AGGACCTGGTAGTGCGAGTG-3′ and 5′-GCGTGTGGCGAAGAA-3′; *col2a1*: 5′- AATGGGCAGAGGTATAAAGATAAGGA-3′ and 5′-CATTCCCAGTGTCACACACACA-3′; *col10a1*: 5′-TTCATCCCATACGCCATAAAG-3′ and 5′-AGCTGGGCCAATATCTCCTT-3′; *col1a1*: 5′-CCTGAGTCAGCAGATTGAGAACA-3′ and 5′-CCAGTACTCTCCGCTCTTCCA-3′, *runx2*: 5′*-*CTGCAAGAAGGCTCTGGCGT-3′ and 5′-CGGTTGGTCTCGGTGGCTGG-3′; *opn*: 5′*-*GGCATTGCCTCCTCCCTC-3′ and 5′-GCAGGCTGTAAAGCTTCTCC-3′; *osx*: 5′-GCAACTGGCTAGGTGGTGGTC-3′ and 5′-GCAAAGTCAGATGGGTAAGTAGGC-3′; *ocn-1*: 5′-CTCTGCTGACCCTGGCTGCG-3′ and 5′-TGAGGCTCCAAGGTAGCGCC-3′; *ocn-2*: 5′- TGACCTCACAGATGCCAAGCCCA-3′ and 5′-AGGGCTCTGGCCACTTACCCA-3′; and *18 s*: 5′-GTAACCCGTTGAACCCCATT-3′ and 5′-CCATCCAATCGGTAGTAGCG-3′.

### Western blot analysis

Knees from WT mice (*n* = 14) and *Mig-6*^*−/−*^mice (*n* = 12) were homogenized in 1 ml of radioimmunoprecipitation assay lysis buffer (50 mM Tris–HCl, 150 mM NaCl, 1 mM ethylenediaminetetraacetic acid, 1 mM ethylene glycol tetraacetic acid, 2 mM sodium orthovanadate, 20 nM sodium pyrophosphate, 1% Triton X-100, 1% sodium deoxycholate, and 0.1% SDS) using a Fast Prep-24 tissue and cell homogenizer. Protein concentration was determined by bicinchoninic acid assay (Thermo Scientific, Rockford, IL, USA). Protein (40 μg) was loaded into the lanes of a 4% to 20% Mini-PROTEAN TGX Precast Gel (Bio-Rad Laboratories, Hercules, CA, USA) and subjected to electrophoresis. Membranes were probed for phospho-EGFR, phospho-p44/42 ERK (pERK), total p44/42 ERK or β-tubulin.

### Enzyme-linked immunosorbent assay

We measured receptor activator of the nuclear factor κB ligand (RANKL) and osteoprotegerin (OPG), which play important roles in the formation of active osteoclasts and can inhibit the RANKL/RANK interaction, respectively [37]. Blood (200 μl) was collected from the orbital sinus of WT mice (*n* = 7) and *Mig-6*^*−/−*^mice (*n* = 5) before surgery (baseline) and 2 and 4 weeks after ligament and meniscus injury according to established animal protocols. Blood was incubated for 2 hours at 37°C and spun down at 12,000 rpm for 10 minutes at 4°C, and serum was collected. OPG and RANKL concentrations were determined using enzyme-linked immunosorbent assay (ELISA) kits from R&D Systems (Minneapolis, MN, USA) according to the manufacturer’s instructions. All samples were assayed in duplicate.

### Statistical analysis

GraphPad Prism software (GraphPad Software, La Jolla, CA, USA) was used for statistical analysis. We confirmed normality of the data sets and used a two-sided unpaired Student’s *t*-test with Welch’s correction for quantitative RT-PCR data. Repeated-measures analysis was used for ELISA data. Two-way analysis of variance with Tukey’s test for multiple comparisons was used for all other quantitative data. We conducted an *F*-test to determine whether variances were significantly different between data sets and performed log transformation on data for which variances were significantly different. *P*-values less than 0.05 were considered statistically significant.

## Results

### Early and severe degeneration of the knee joint after injury in *Mig-6*^*−/−*^mice

**Figure 1:**
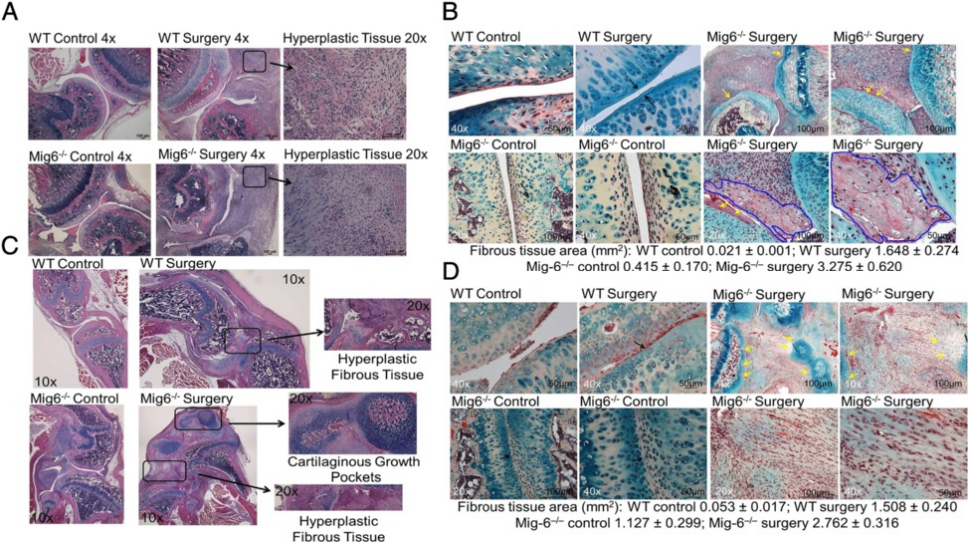
**Animals that were used for micro–computed tomography were also used for histological analysis. (A)** Hematoxylin and eosin (H&E)–stained sections of control and surgically treated knees from wild-type (WT) and inactivated mitogen-inducible gene 6 (*Mig-6*^*−/−*^) mice at 2 weeks after surgery (original magnification, 4×). Boxed regions are shown at higher magnification (original magnification, 20×). **(B)** Pentachrome-stained sagittal sections of control and surgically treated knees from WT and *Mig-6*^*−/−*^mice at the 2-week time point. The black arrows point to joint space narrowing, the yellow arrows point to cartilage degradation and fibrous tissue is outlined in blue. Muscle is stained red, cartilage blue, fibrin bright red, and reticular yellow. Fibrous tissue area was quantified using ImageJ software (data are mean ± SE). **(C)** H&E-stained sections of control and surgically treated knees from WT and *Mig-6*^*−/−*^mice at 4 weeks (original magnification, 4×). Boxed regions are shown at higher magnification (original magnification, 20×). **(D)** Pentachrome-stained sagittal sections of control and surgically treated knees from WT and *Mig-6*^*−/−*^mice at the 4-week time point. The black arrow points to joint space narrowing. Yellow arrows point to cartilage degradation. Fibrous tissue within the joint space of *Mig-6*^*−/−*^surgically treated knees is shown at 20× and 40× original magnification and was quantified using ImageJ software (mean ± SE).

Two weeks after ligament and meniscal transections, hyperplastic disorganized fibrous tissue formed in the knee joint space of *Mig-6*^*−/−*^and WT mice. We observed a significantly greater fibrous tissue area and greater staining for fibrin in surgically treated knees of *Mig-6*^*−/−*^mice (*P* < 0.001) and WT mice (*P* < 0.05) relative to respective control knees (Figure 1A and 1B) The area of fibrous tissue in *Mig-6*^*−/−*^surgically treated knees was significantly greater than that in WT control (*P* < 0.001) or surgically treated knees (*P* < 0.05) (Figure 1A and 1B).

Four weeks after surgery, the hyperplastic fibrous tissue region was significantly greater in the surgically treated knees of *Mig-6*^*−/−*^mice (*P* < 0.001) and WT mice (*P* < 0.01) than in the knees of respective control mice (Figure 1C and 1D). Surgically treated knees of *Mig-6*^*−/−*^mice included cartilaginous pockets in areas outside the articulating surface (Figure 1C). We also qualitatively observed cartilage degradation in surgically treated knees from *Mig-6*^*−/−*^mice and joint space narrowing in surgically treated knees from WT animals (Figure 1D). The fibrous tissue area was significantly greater for both knees from *Mig-6*^*−/−*^mice relative to the respective knees from WT animals (*P* < 0.0001) (Figure 1C and 1D).

Four weeks after surgery, joint morphology was qualitatively similar between male and female mice of each genotype (Additional file 1: Figure S1A). Therefore, samples were pooled for all quantitative analyses. Articular cartilage thickness was significantly lower in *Mig-6*^*−/−*^ (*P* < 0.001) and WT (*P* < 0.05) surgically treated knees relative to respective control knees (WT control = 0.265 ± 0.023 mm, WT surgically treated = 0.187 ± 0.015 mm, *Mig-6*^*−/−*^control = 0.236 ± 0.031 mm and *Mig-6*^*−/−*^ = surgically treated = 0.100 ± 0.027 mm). Articular cartilage thickness in surgically treated knees from *Mig-6*^*−/−*^mice was significantly lower than that in WT surgically treated mice (*P* < 0.05) and WT control mice knees (*P* < 0.0001). The histological score, indicative of disease severity, increased significantly with surgery for both *Mig-6*^*−/−*^mice (*P* < 0.01) and WT mice (*P* < 0.01) and was significantly higher for *Mig-6*^*−/−*^knees than for WT control knees (*P* < 0.05) (WT control = 1.1 ± 0.3, WT surgically treated = 4.8 ± 0.3, *Mig-6*^*−/−*^control = 2.4 ± 0.6 and *Mig-6*^*−/−*^surgically treated = 5.3 ± 0.3).

**Figure 2:**
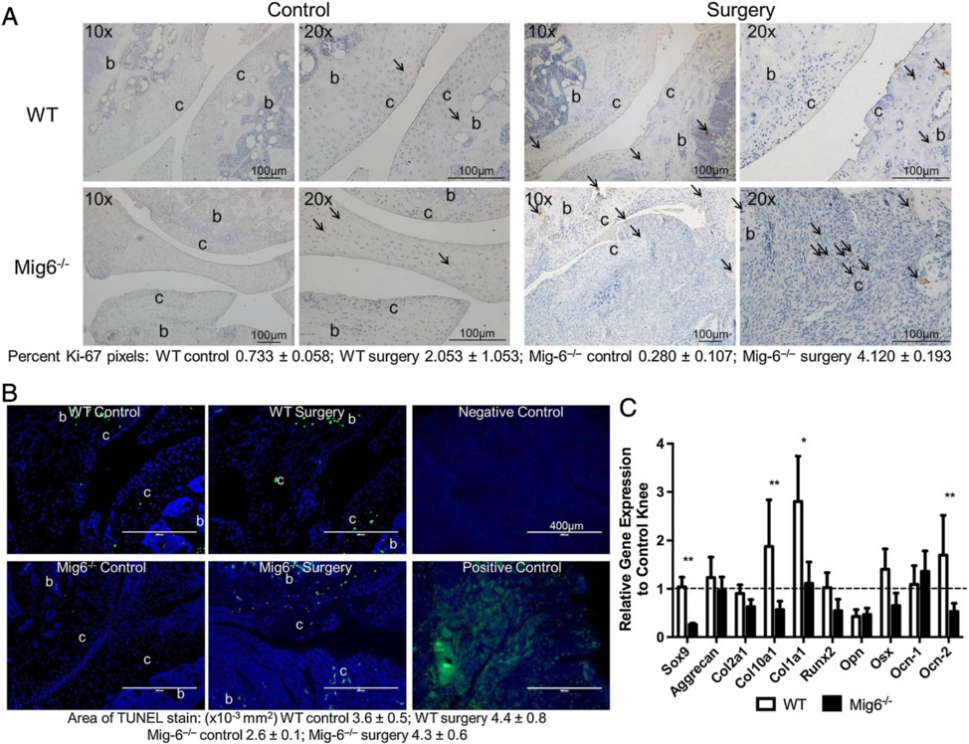
**Cartilage and subchondral bone cell analysis. (A)** Ki67 stain (brown) of knee tissue from wild-type (WT) and inactivated mitogen-inducible gene 6 (*Mig-6*^*−/−*^) mice at 4 weeks after surgery. Arrows point to stained cells and cartilage (c) and subchondral bone (b) are labeled. For WT mice, osteoblasts are stained in the subchondral bone and chondrocytes are stained in the cartilage. For *Mig-6*^*−/−*^mice, osteoblasts are stained in the subchondral bone, chondrocytes are stained in the control knee cartilage and both fibroblasts and chondrocytes are stained in the surgically treated knee cartilage. **(B)** Terminal deoxynucleotidyl transferase deoxyuridine triphosphate nick-end labeling (TUNEL) stain of WT and *Mig-6*^*−/−*^tissues at 4 weeks postsurgery. Apoptotic cells are stained green, and nuclei are stained blue. c = cartilage and b = subchondral bone. For WT mice, osteoblasts are stained in the subchondral bone and chondrocytes are stained in the cartilage. For *Mig-6*^*−/−*^mice, osteoblasts are stained in the subchondral bone, chondrocytes are stained in the control knee cartilage and both fibroblasts and chondrocytes are stained in the surgically treated knee cartilage. ImageJ software was used to quantify the area of positive TUNEL staining (mean ± SE). **(C)** RT-PCR data for WT and *Mig-6*^*−/−*^surgically treated knees (relative to control knees) at 4 weeks after surgery, expressed as mean ± SE. **P < 0.01 between genotypes and *P < 0.05 between genotypes. The amplification of gene products was determined by the incorporation of SYBR Green I Nucleic Acid Gel Stain (Life Technologies, Carlsbad, CA, USA) and normalized to *18 s* using the 2^ΔΔ−Ct^ method. Col10a1: Collagen, type X, α1; Col1a1: Collagen, type I, α1; Col2a1: Collagen, type II, α1; Ocn-1: Osteocalcin variant 1; Ocn-2: Osteocalcin variant 2; Opn: Osteopontin; Osx: Osterix; Runx2: Runt-related transcription factor 2.

Cell proliferation and apoptosis were affected by injury and loss of *Mig-6*. There was a significant increase (*P* < 0.001) in the percentage of Ki-67-stained pixels, indicative of cell proliferation, in *Mig-6*^*−/−*^surgically treated knees relative contralateral control knees (Figure 2A). The percentage of proliferating cells was significantly greater (*P* < 0.05) in surgically treated knees from *Mig-6*^*−/−*^mice than in surgically treated knees from WT animals (Figure 2A). *Mig-6*^*−/−*^mice had a significant increase (*P* < 0.01) in the area of the TUNEL staining in surgically treated knees relative to control knees, but there was no difference between the genotypes (Figure 2B).

We observed differences in chondrogenic and osteogenic markers in knees from *Mig-6*^*−/−*^and WT mice. Of chondrogenic and osteogenic markers in *Mig-6*^*−/−*^surgically treated knees (relative to control knees), *Sox9*, *Col10a1*, *Runx2*, *Opn* and *Ocn-2* were downregulated 5-, 1.8-, 1.9-, 2.1- and 2-fold, respectively, relative to controls (Figure 2C). In WT mice, *Col10a1*, *Col1a1* and *Ocn-2* were upregulated 1.9-, 2.8- and 1.7-fold, respectively, and *Opn* was downregulated 2.3-fold (Figure 2C). Relative *Sox9* expression was significantly lower in *Mig-6*^*−/−*^knees than in WT (*P* < 0.01). Expression of *Col10a1* (*P* < 0.01), *Col1a1* (*P* < 0.05) and *Ocn-2* (*P* < 0.01) was significantly lower as well (Figure 2C).

### Changes in bone structure after injury and loss of *Mig-6*

**Figure 3:**
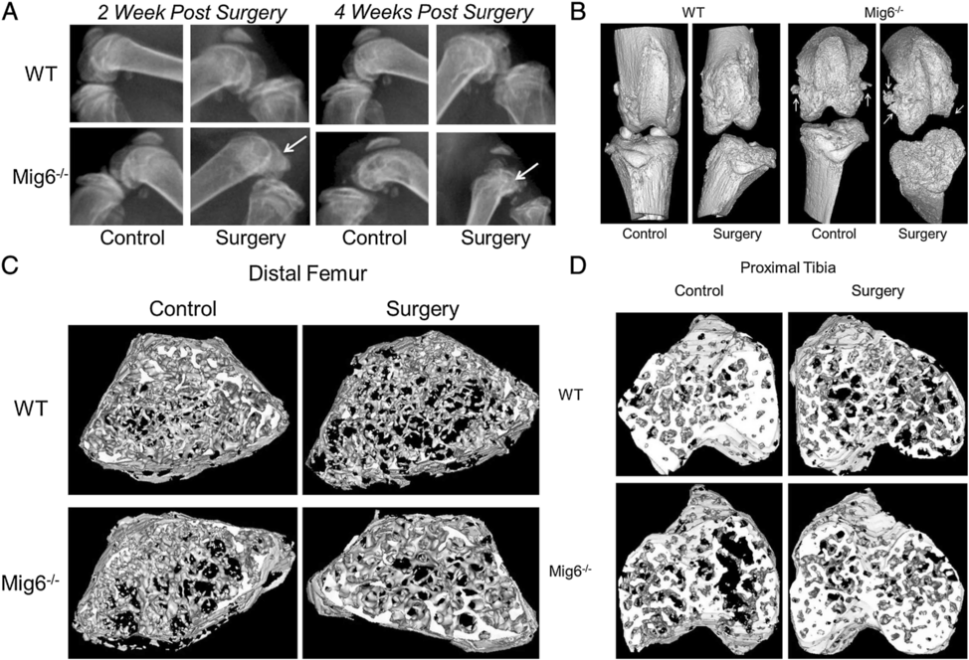
**Three-dimensional reconstruction images were created for use in qualitative analysis.** Images were reconstructed using Mimics software (Materialise, Plymouth, MI, USA). **(A)** X-rays of control and surgically treated knees from wild-type (WT) and inactivated mitogen-inducible gene 6 (*Mig-6*^*−/−*^) mice 2 and 4 weeks after surgery. Arrows point to subchondral bone erosion. **(B)** Three-dimensional reconstructed isosurfaces of knees from WT and *Mig-6*^*−/−*^mice at 4 weeks after surgery. Arrows point to erosion and fragments in the subchondral bone. **(C)** Transverse view of three-dimensional reconstructed isosurfaces of trabecular bone from the distal femurs of WT and *Mig-6*^*−/−*^knees 4 weeks after surgery. **(D)** Transverse view of three-dimensional reconstructed isosurfaces of trabecular bone from the proximal tibia of knees from WT and *Mig-6*^*−/−*^knees 4 weeks after surgery.

Femoral condyle bone erosion was evident by X-ray 2 weeks after surgery in *Mig-6*^*−/−*^mice, and deterioration of the femoral subchondral bone increased in severity qualitatively at 4 weeks (Figure 3A). Subchondral bone damage was qualitatively observed in three-dimensional, reconstructed micro-CT images of surgically treated knees from *Mig-6*^*−/−*^mice at 4 weeks (Figure 3B). Relative to WT control knees, we observed differences in trabecular bone geometry in the distal femur and proximal tibia of WT surgically treated knees and in knees from *Mig-6*^*−/−*^mice (Figure 3C and 3D).

In surgically treated knees relative to control knees, tibial trabecular bone density and the number of trabeculae decreased significantly (*P* <0.05), but the separation distance between trabeculae increased significantly (*P* < 0.01), for *Mig-6*^*−/−*^mice (Table 1). The thickness of trabeculae in the distal femur (*P* < 0.05) and proximal tibia (*P* < 0.01) were significantly higher in surgically treated knees of *Mig-6*^*−/−*^mice than in control knees, and the distal femur bone volume fraction (ratio of the segmented bone volume to the total volume of the region of interest) was significantly lower in WT surgically treated knees than in control knees (*P* < 0.05) (Table 1). In the proximal tibia of *Mig-6*^*−/−*^surgically treated knees, trabecular thickness (*P* < 0.001), trabecular BMD (*P* < 0.01) and number of trabeculae (*P* < 0.05) were significantly lower than in the WT surgically treated knees (Table 1). Femur trabecular thickness was significantly higher in the surgically treated knees of *Mig-6*^*−/−*^mice than in WT surgically treated knees (*P* < 0.001) (Table 1). Both tibia trabecular BMD (*P* < 0.05) and the number of femoral trabeculae (*P* < 0.01) were significantly lower in control knees of *Mig-6*^*−/−*^mice than in WT control knees, and the distance between trabeculae was significantly larger for control knees of *Mig-6*^*−/−*^mice than for WT control knees in this region (*P* < 0.05) (Table 1). The effect of surgery on trabecular thickness within the tibia and femur was significantly different between genotypes (*P* < 0.01 for interaction).

**Table 1:** **Tibia and femur trabecular bone micro-CT data**^**a**^

**Bone**	**WT**		***Mig-6***^***−/−***^	
	**Control**	**Surgery**	**Control**	**Surgery**
Tibia				
Trabecular BMD (g/ml)	0.579 ± 0.010	0.528 ± 0.006^b^	0.524 ± 0.028^c^	0.459 ± 0.009^b,d^
Trabecular thickness (mm)	0.022 ± 0.000	0.022 ± 0.001	0.020 ± 0.001	0.021 ± 0.001^e,f^
Number of trabeculae	5.918 ± 0.280	5.437 ± 0.405^b^	4.395 ± 0.744	2.545 ± 0.187^g,h^
Separation distance between trabeculae (mm)	0.138 ± 0.012	0.139 ± 0.004	0.242 ± 0.044	0.289 ± 0.063^e^
BV/TV (%)	12.473 ± 0.483	13.042 ± 0.340	8.385 ± 1.780	10.670 ± 3.765
Femur				
Trabecular BMD (g/ml)	0.590 ± 0.011	0.579 ± 0.015	0.551 ± 0.019	0.533 ± 0.022
Trabecular thickness (mm)	0.025 ± 0.001	0.023 ± 0.001	0.024 ± 0.002	0.031 ± 0.001^b,f^
Number of trabeculae	6.214 ± 0.632	4.866 ± 0.419	3.716 ± 0.093^i^	4.573 ± 0.545
Separation distance between trabeculae (mm)	0.124 ± 0.009	0.158 ± 0.023	0.225 ± 0.040^c^	0.190 ± 0.033
BV/TV (%)	16.502 ± 1.011	10.893 ± 1.069^b^	11.747 ± 2.764	14.499 ± 2.678

^a^BMD: Bone mineral density; BV/TV: Bone volume fraction (ratio of the segmented bone volume to the total volume of the region of interest); *Mig-6*^*−/−*^: Inactivated mitogen-inducible gene 6; WT: Wild type. Animals that were X-rayed were also used for micro-CT analysis. Data are expressed as mean ± SE. ^b^*P* < 0.05 between control and surgery. ^c^*P* < 0.05 between genotypes for control knees. ^d^*P* < 0.01 between genotypes for surgically treated knees. ^e^*P* < 0.01 between control and surgery. ^f^*P* < 0.001 between genotypes for surgically treated knees. ^g^*P* < 0.001 between control and surgery. ^h^*P* < 0.05 between genotypes for surgically treated knees. ^i^*P* < 0.01 between genotypes for control knees.

### Increased inflammatory cells and osteoclasts in *Mig-6*^*−/−*^mice

**Figure 4:**
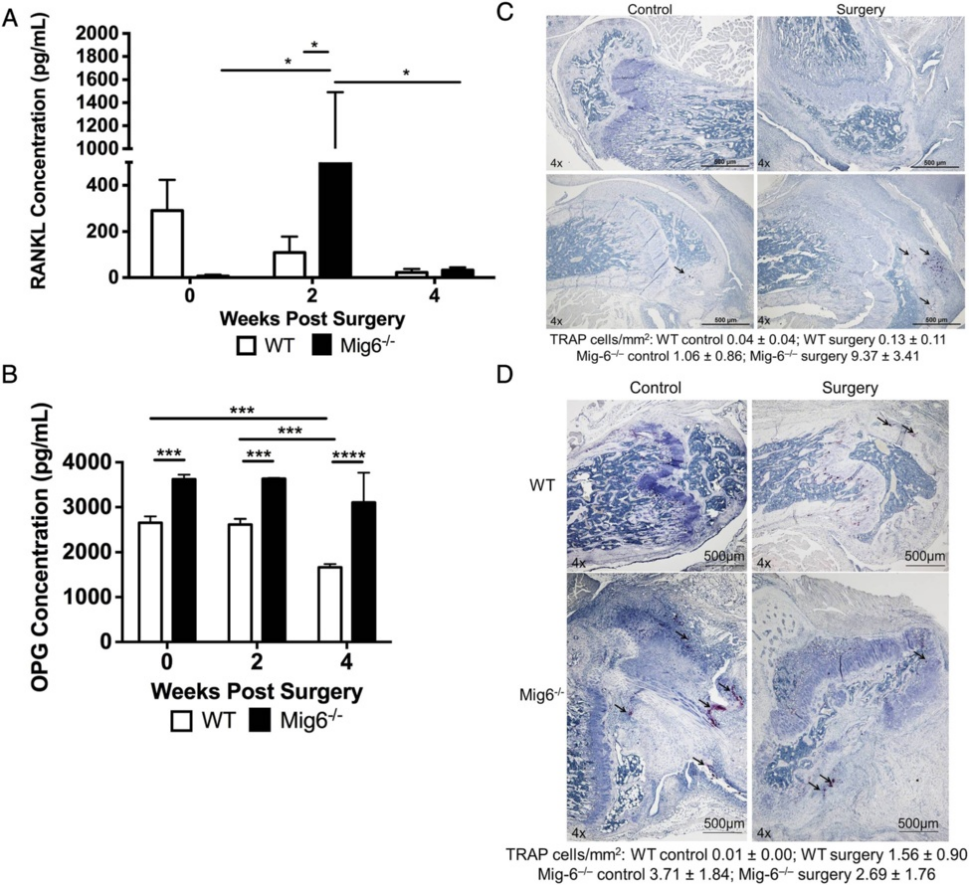
**Mice used for micro-CT analysis were also used for enzyme-linked immunosorbent assay measurements. (A)** Serum receptor activator of nuclear factor κB ligand (RANKL) concentration for wild-type (WT) and inactivated mitogen-inducible gene 6 (*Mig-6*^*−/−*^) mice expressed as mean ± SE. *P < 0.05. **(B)** Serum osteoprotegerin (OPG) concentration for WT and *Mig-6*^*−/−*^mice expressed as mean ± SE. ***P < 0.001 and ****P < 0.0001. **(C)** Arrows point to tartrate-resistant acid phosphatase (TRAP)-positive cells (pink) in the subchondral bone of knees from WT and *Mig-6*^*−/−*^mice 2 weeks after surgery. The number of TRAP-stained cells was counted manually and normalized to the tissue area (mean ± SE). **(D)** Arrows point to TRAP-positive cells (pink) in the subchondral bone of knees from WT and *Mig-6*^*−/−*^mice 4 weeks after surgery. The number of TRAP-stained cells was counted manually and normalized to the tissue area (mean ± SE).

Serum RANKL concentration was significantly greater in *Mig-6*^*−/−*^mice 2 weeks after surgery relative to the concentrations in *Mig-6*^*−/−*^mice at baseline and at 4 weeks after surgery and relative to the concentration for WT animals at 2 weeks after surgery (*P* < 0.05) (Figure 4A). The effects of genotype on RANKL concentration over time were significantly different (*P* < 0.05 for interaction). For WT mice, OPG concentration was significantly lower (*P* < 0.001) at 4 weeks after surgery relative to the concentrations at 2 weeks after surgery and at baseline (Figure 4B). Serum OPG concentration was significantly greater for *Mig-6*^*−/−*^mice at baseline (*P* < 0.001) and at 2 weeks after surgery (*P* < 0.001) and at 4 weeks after surgery (*P* < 0.0001) relative to the concentrations for WT animals (Figure 4B), but there were no significant differences in the RANKL/OPG ratios between genotypes or time points (Additional file 1: Figure S1B).

**Figure 5:**
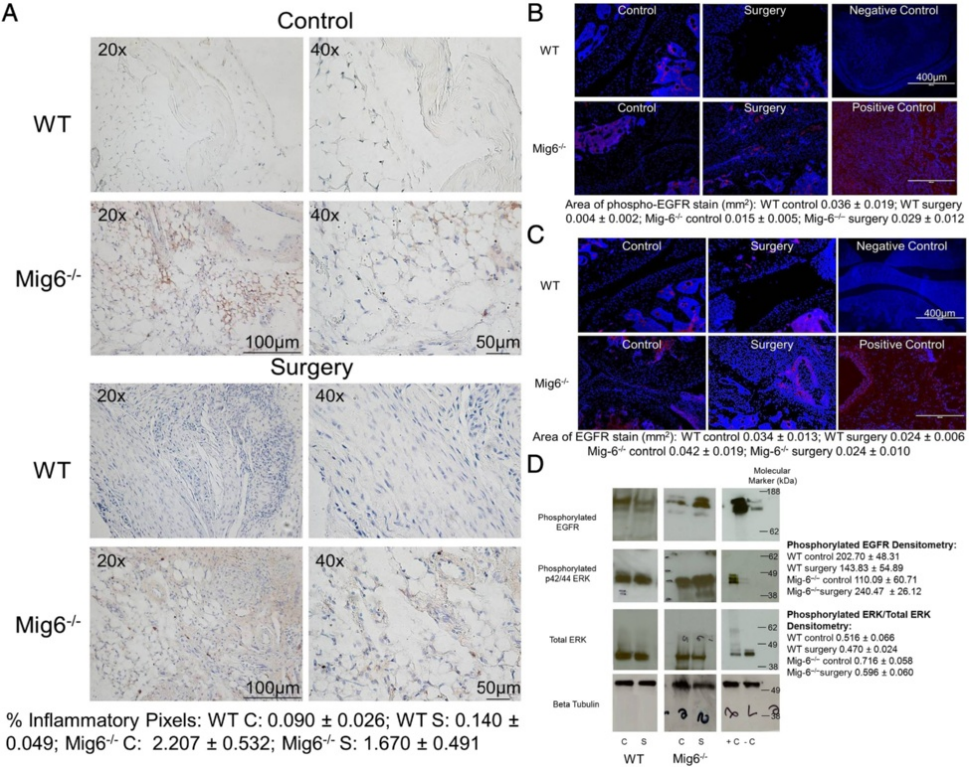
**Inflammation and epidermal growth factor receptor analysis. (A)** Sagittal sections of knees from wild-type (WT) and inactivated mitogen-inducible gene 6 (*Mig-6*^*−/−*^) mice 4 weeks after surgery stained for monocytes, neutrophils and macrophages (brown). C: control knee and S: surgery knee. **(B)** Phosphorylated epidermal growth factor receptor (phospho-EGFR) staining (red) of knees from WT and *Mig-6*^*−/−*^mice counterstained with 4′,6-diamidino-2-phenylindole (DAPI blue) (original magnification, 10×) 4 weeks after surgery. ImageJ software was used to quantify the area of phospho-EGFR staining (mean ± SE). **(C)** Total EGFR staining (red) of knees from WT and *Mig-6*^*−/−*^mice counterstained with DAPI (blue) (original magnification, 10×) 4 weeks after surgery. ImageJ software was used to quantify the area of total EGFR staining (mean ± SE). **(D)** Western blots from control (C) and surgery (S) knees isolated from WT and *Mig-6*^*−/−*^mice probed for phospho-EGFR, phosphorylated extracellular signal-regulated kinase (phospho-ERK), total ERK and β-tubulin. Densitometry (mean ± SE) was determined using ImageJ software. The positive (+C) and negative control (−C) lysates were protein extracts from calvarial osteoblasts isolated from 3-day-old WT mice subjected to either 60 minutes of 2 Pa of fluid shear stress or no mechanical stimulation. Gel lane numbers are written on some of the blots.

We observed significantly greater TRAP-positive cells normalized to tissue area in the subchondral bone of surgically treated knees from *Mig-6*^*−/−*^mice at 2 weeks relative to *Mig-6*^*−/−*^control knees (*P* < 0.01) and relative to knees from WT animals (*P* < 0.001) (Figure 4C). We found significantly greater TRAP-positive cells in the subchondral bone of control knees from *Mig-6*^*−/−*^mice at 4 weeks relative to control knees from WT mice (Figure 4D). We did not find monocytes, neutrophils or macrophages in WT or *Mig-6*^*−/−*^mice 2 weeks after surgery (data not shown). The percentage of stained pixels for monocytes, neutrophils and macrophages was significantly greater in control and surgically treated knees of *Mig-6*^*−/−*^mice than in WT controls (*P* < 0.001 and *P* < 0.01, respectively) or WT surgically treated knees (*P* < 0.01 and *P* < 0.01, respectively) (Figure 5A).

### Differences in alteration of phosphorylated epidermal growth factor receptor protein after injury

The area of phospho-EGFR staining was significantly lower at 4 weeks in control knees from *Mig-6*^*−/−*^mice relative to surgically treated knees (*P* < 0.01) (Figure 5B). The area of phospho-EGFR staining was significantly lower at 4 weeks in surgically treated knees from WT mice relative to control knees (*P* < 0.0001) and also to surgically treated knees from *Mig-6*^*−/−*^mice (*P* < 0.05) (Figure 5B). The effect of surgery on phospho-EGFR staining in WT and *Mig-6*^*−/−*^mice at 4 weeks was significantly different (*P* < 0.0001 for interaction).

There were no significant differences in the area of EGFR staining between control and surgically treated knees from WT and *Mig-6*^*−/−*^mice at 4 weeks or between genotypes (Figure 5C). Phospho-EGFR densitometry was significantly lower at 4 weeks in surgically treated knees from WT mice relative to control knees (*P* < 0.05) and also to surgically treated knees from *Mig-6*^*−/−*^mice (*P* < 0.0001) (Figure 5D). Phospho-EGFR densitometry was significantly lower at 4 weeks in control knees from *Mig-6*^*−/−*^mice relative to surgically treated knees (*P* < 0.0001) (Figure 5D). There were no significant differences in pERK densitometry or total ERK densitometry between control and surgically treated knees from WT and *Mig-6*^*−/−*^mice at 4 weeks or between genotypes (Figure 5D and Additional file 2: Table S1). The phospho-ERK densitometry normalized to total ERK densitometry was significantly lower in control knees (*P* < 0.01) and surgically treated knees (*P* < 0.0001) from WT mice relative to control knees from *Mig-6*^*−/−*^mice (Figure 5D).

## Discussion

We found exacerbated and accelerated cartilage and subchondral bone damage in young *Mig-6*^*−/−*^mice after ligament and meniscal trauma, as well as increased EGFR phosphorylation. Increased cell proliferation has been reported in *Mig-6*^*−/−*^mice, and reduced proliferation has been reported in cells overexpressing *Mig-6* and treated with hepatocyte growth factor [31,38]. We found no differences between genotypes in terms of control knee cell proliferation, but we observed a greater number of proliferating cells and staining for phospho-EGFR in surgically treated knees from *Mig-6*^*−/−*^mice (relative to their control knees and to surgically treated knees from WT mice), which likely contributes to increased cell growth. Despite increased proliferation, *Sox9* gene expression was downregulated in *Mig-6*^*−/−*^surgically treated knees, which could be due to reduced articular cartilage.

We did not detect consistent increases in phospho-ERK, total ERK or phospho-EGFR protein levels for both knees from *Mig-6*^*−/−*^mice relative to WT, but these proteins can be influenced by other pathways that balance EGFR signaling. EGFR signaling is maintained by the endocytic pathway, which is crucial for the spatial localization of the EGF–EGFR complex needed to activate downstream effectors [39]. EGF binding can cause the EGF–EGFR complex to internalize and traverse the endocytic pathway, moving from clathrin-coated vesicles through endosomes to lysosomes for degradation [39]. This process, though it was not examined in this study, could sequester EGFR from downstream effectors in addition to removing the receptor from the cell surface, dephosphorylating the receptor or targeting the ligand–receptor complex for degradation [39].

Investigators in several studies have highlighted the influence of *Mig-6* and EGFR on apoptosis. Pharmacological compounds can induce apoptosis in lung and breast cancer cells by inhibiting the EGFR pathway, and recent work has demonstrated that *Mig-6* actively senses EGF deprivation to directly activate proapoptotic c-Abl in murine epithelial cells [40-42]. *Mig-6* can trigger apoptosis as well as exacerbate endoplasmic reticulum stress-induced β-cell death [43]. Apoptosis inhibition in MCF7 human breast cancer cells with exogenous overexpression of *Mig-6* has been shown, but increased cleaved caspase 3 immunostaining in lungs from *Mig-6*^*−/−*^mice has also been reported [31,44]. In our present study, we observed an increase in apoptotic cells in the surgically treated knees from *Mig-6*^*−/−*^mice relative to control knees, which may be attributed to increased EGFR phosphorylation and also to damage in the articular cartilage and underlying subchondral bone.

EGFR signaling has been linked to inflammation, but limited data exist regarding the effect of *Mig-6* on inflammation. Histological reports show staining for EGFR in the bronchial epithelium, glands, smooth muscle cells and basement membranes of chronically inflamed asthmatic airways, and EGFR inhibition can reduce the number of inflammatory cells, as well as interleukin 4 and interleukin 13 concentrations, in bronchoalveolar lavage fluid [45,46]. EGFR inhibition can also reduce eosinophil recruitment in the lungs as well as airway hyperresponsiveness. The concentrations of serum and joint EGF and EGFR are significantly higher in RA patients than in healthy populations, and the thymuses of *Mig-6*^*−/−*^mice are larger than normal size; however, significant numbers of inflammatory cells were not observed in previous studies of *Mig-6*^*−/−*^joints [26,46]. *Mig-6* can be transcriptionally induced by hormones and growth factors as well by stresses such as mechanical strain and impact [47,48]. In *Mig-6*^*−/−*^mice, we found increased numbers of inflammatory cells, which may occur naturally in younger *Mig-6*^*−/−*^mice prior to observation of significant spontaneous disease and could also be a compensatory response to the stresses placed on the surgically treated knee joint by the altered mechanical environment induced by ligament and meniscus injury [49].

Relative to WT animals, we observed some impairment to trabecular bone in the control and surgically treated knees of *Mig-6*^*−/−*^mice, which could reduce the mechanical integrity of the knee joints [50]. There was an increase in trabecular thickness with surgery in *Mig-6*^*−/−*^mice, which may be a result of skeletal adaptation and an attempt to compensate for joint damage and/or increased EGFR phosphorylation [13]. There is also evidence that EGFR signaling is involved in osteoclast formation and that it may have functional importance for osteoclast differentiation and survival. Thus, increased EGFR phosphorylation in surgically treated knees of *Mig-6*^*−/−*^mice could contribute to the observed subchondral bone resorption and increased RANKL [36].

It has been suggested that the impact of *Mig-6* might manifest only during dynamic processes such as development or injury repair [31]. EGFR is activated in response to a wounding event, and signaling pathways downstream from EGFR are central in regulating distinct stages of wound healing [51]. Together, the inactivation of *Mig-6* and increased EGFR phosphorylation in surgically treated knees of mutant mice may increase inflammation and thus apoptosis, leading to reduced articular cartilage thickness. Reduced cartilage thickness in *Mig-6*^*−/−*^mice could further increase the loads on the joint, causing additional damage to the underlying subchondral bone. Despite increased cell proliferation (perhaps in an enhanced attempt to repair damage), the joint integrity in *Mig-6*^*−/−*^mice remains compromised because of undirected cell growth. Therefore, *Mig-6*^*−/−*^mice develop rapid and more severe cartilage and subchondral bone damage after ligament and meniscus injury.

Four weeks after surgery, we observed joint space narrowing in control knees from *Mig-6*^*−/−*^mice that was comparable to that previously reported in 1.5-month-old *Mig-6*^*−/−*^animals, but we did not observe joint space narrowing in WT control knees and therefore attribute this phenomenon to early-stage joint disease associated with loss of function in *Mig-6*^*−/−*^animals. Fifty percent of *Mig-6*^*−/−*^mice die before adolescence, likely because of impairments in pulmonary development and vascularization of the lungs [31]. The limited survival of adult *Mig-6*^*−/−*^mice enabled us to study only early changes to cartilage and subchondral bone after trauma to ligaments and the meniscus in young mice. Several cell types are found in the knee joint under normal and pathological conditions. Synovial lining cells produce lubricin and hyaluronic acid, which help to protect and maintain the integrity of the cartilage surface [8]. Fibroblasts, macrophages and lymphocytes participate in synovial hyperplasia and inflammation, and osteoblasts, osteoclasts and chondrocytes, respectively, form and resorb bone and maintain cartilage [3,8,52]. In our present study, we were able to observe the effect of inactivation of *Mig-6* in all cell types involved in joint maintenance after ligament and meniscal damage, but we did not completely elucidate the exact mechanism of action of *Mig-6* in joints. Our study opens the door for future work focused on dissecting the exact mechanism of Mig-6 and EGFR signaling in joint damage after ligament and meniscus injury.

## Conclusion

Phospho-EGFR was lower in surgically treated knees from WT mice relative to controls, and joint damage was less severe in these animals relative to *Mig-6*^*−/−*^mice, which conversely had increased phospho-EGFR in surgically treated knees relative to controls. There could be a threshold of EGFR signaling required for maximum therapeutic effect after trauma to the ligaments and meniscus, and attenuation of *Mig-6* could be a strategic tool for finely altering EGFR signaling and reducing cartilage and subchondral bone damage after this type of injury.

## Abbreviations

Akt: Protein kinase B; ANOVA: Analysis of variance; BMD: Bone mineral density; BTC: Betacellulin; BV/TV: Bone volume fraction (ratio of the segmented bone volume to the total volume of the region of interest); Col10a1: Collagen type X, α1; Col1a1: Collagen type I, α1; Col2a1: Collagen type II, α1; DAPI: 4′,6-diamidino-2-phenylindole; ECM: Extracellular matrix; EDTA: Ethylenediaminetetraacetic acid; EGF: Epidermal growth factor; EGFR: Epidermal growth factor receptor; ELISA: Enzyme-linked immunosorbent assay; ER: Endoplasmic reticulum; ERK: Extracellular signal-regulated kinase (p44/42 mitogen-activated protein kinase); ERRFI1 and ERBB: receptor feedback inhibitor 1; HB-EGF: Heparin-binding epidermal growth factor; IF: Immunofluorescence; IHC: Immunohistochemistry; JNK: c-Jun N-terminal kinase; KO: Knockout; MAP kinase: Mitogen-activated protein kinase; MIG-6: Mitogen-inducible gene 6; NaCl: Sodium chloride; OA: Osteoarthritis; Ocn-1: Osteocalcin variant 1; Ocn-2: Osteocalcin variant 2; OPG: Osteoprotegerin; Opn: Osteopontin; Osx: Osterix; pERK and phospho-ERK: Phosphorylated extracellular signal-regulated kinase (phosphorylated p44/42 mitogen-activated protein kinase); Phospho-EGFR: Phosphorylated epidermal growth factor receptor; PI3kinase: Phosphatidylinositide 3-kinase; RA: Rheumatoid arthritis; RALT: Receptor-associated late transducer; RANK: Receptor activator of nuclear factor κB; RANKL: Receptor activator of nuclear factor κB ligand; Runx2: Runt-related transcription factor 2; TFG-α: Transforming growth factor α; TRAP: Tartrate-resistant acid phosphatase; TUNEL: Terminal deoxynucleotidyl transferase deoxyuridine triphosphate nick-end labeling; WT: Wild type.

## Competing interests

The authors declare that they have no competing interests.

## Authors’ contributions

DMJ, KDL, and BOW designed the research. DMJ, KDL, EMV and DH performed the research. DMJ, KDL and EMV analyzed the data. DMJ wrote the paper, which was edited by KDL, EMV, DH and BOW. All authors read and approved the final manuscript.

## Authors’ information

DMJ is a research fellow in internal medicine, pulmonary/critical care and immunology at the University of Michigan and a former fellow at the Laboratory of Cell Signaling and Carcinogenesis, Van Andel Research Institute. BOW is an associate professor and director of the Center for Skeletal Disease and Tumor Metastasis, as well as the head of the Laboratory of Cell Signaling and Carcinogenesis, at the Van Andel Research Institute.

## Supplementary Material

Additional file 1: Figure S1 **(A)** Pentachrome-stained sagittal sections of control and surgically treated knees from male and female wild-type (WT) and inactivated mitogen-inducible gene 6 (*Mig-6*^*−/−*^) mice 4 weeks after surgery. Muscle (red), cartilage (blue), fibrin (bright red) and reticular fibers (yellow) are shown. Original magnification, 4×. **(B)** Serum RANKL (receptor activator of nuclear factor κB ligand) concentration normalized to osteoprotegerin concentration for WT and *Mig-6*^*−/−*^mice expressed as mean ± SE. Click here for file

Additional file 2: Table S1 Western blot densitometry. Click here for file
