# Supplementary figures and images for: Accelerated and increased joint damage in young mice with global inactivation of mitogen-inducible gene 6 after ligament and meniscus injury
Source: Arthritis Res Ther. 2014 Mar 27;16(2):R81. doi: 10.1186/ar4522 (PMC4060238; doi:10.1186/ar4522)

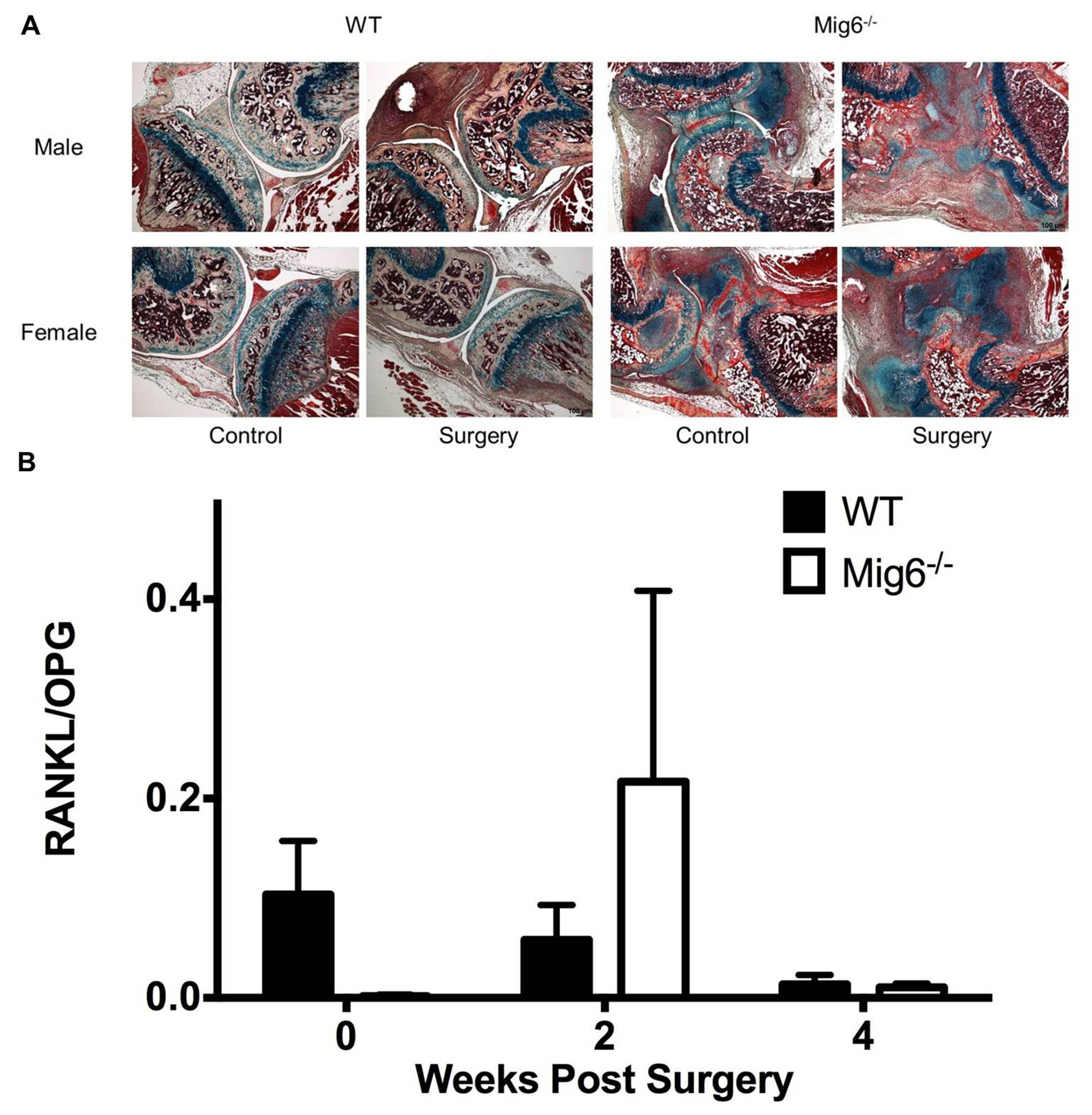

Supplement: Additional file 1: Figure S1 — (A) Pentachrome-stained sagittal sections of control and surgically treated knees from male and female wild-type (WT) and inactivated mitogen-inducible gene 6 (Mig-6−/−) mice 4 weeks after surgery. Muscle (red), cartilage (blue), fibrin (bright red) and reticular fibers (yellow) are shown. Original magnification, 4×. (B) Serum RANKL (receptor activator of nuclear factor κB ligand) concentration normalized to osteoprotegerin concentration for WT and Mig-6−/−mice expressed as mean ± SE. [file ar4522-S1.jpeg]
