# Supplementary material for: Accelerated and increased joint damage in young mice with global inactivation of mitogen-inducible gene 6 after ligament and meniscus injury
Source: Arthritis Res Ther. 2014 Mar 27;16(2):R81. doi: 10.1186/ar4522 (PMC4060238; doi:10.1186/ar4522)
Supplement: Additional file 2: Table S1 — Western blot densitometry. [file ar4522-S2.doc]

**Additional file 2: Table S1:** Western Blot Densitometry^a^

| ^a^ Data (mean ± SE) determined with Image J software | WT | | *Mig6^–/–^* | |
| --- | --- | --- | --- | --- |
|  | Control | Surgery | Control | Surgery |
| Phospho-ERK | 90.7 ± 14.0 | 85.1 ± 9.8 | 122.7 ± 20.7 | 101.5 ± 15.0 |
| Total ERK | 187.2 ± 6.5 | 190.7 ± 10.2 | 178.4 ± 12.5 | 183.0 ± 10.8 |
